# Assessing the quality of reports about randomized controlled trials of scalp acupuncture treatment for vascular dementia

**DOI:** 10.1186/s13063-017-1945-0

**Published:** 2017-05-02

**Authors:** Young-Nim You, Myung-Rae Cho, Ju-Hyung Park, Gwang-Cheon Park, Min-Yeong Song, Jin-Bong Choi, Chang-Su Na, Jae-Young Han, Jeong-cheol Shin, Jae-Hong Kim

**Affiliations:** 1 Clinical Research Center, DongShin University Gwangju Oriental Hospital, Gwangju City, Republic of Korea; 2 Department of Acupuncture & Moxibustion Medicine, College of Korean Medicine Dong-Shin University, Naju City, Republic of Korea; 3 Department of Korean Rehabilitation Medicine, College of Korean Medicine Dong-Shin University, Naju City, Republic of Korea; 4 Department of Meridian and Acupoint, College of Korean Medicine Dong-Shin University, Naju City, Republic of Korea; 5 Department of Physical & Rehabilitation Medicine, Chonnam National University Medical School & Hospital, Gwangju City, Republic of Korea

**Keywords:** Randomized controlled trials, Scalp acupuncture, Vascular dementia, CONSORT, STRICTA

## Abstract

**Background:**

This study aimed to evaluate the quality of reports about randomized controlled trials (RCTs) of scalp acupuncture (SA) for the treatment of vascular dementia (VD).

**Method:**

A systematic search of reports published through to December 2015 was performed in eight databases. The quality of RCTs that used SA as an intervention for VD was evaluated based on the 2010 Consolidated Standards for Reporting of Trials (CONSORT) and 2010 Standards for Reporting Interventions in Controlled Trials of Acupuncture (STRICTA) guidelines. Thirteen items from the CONSORT guideline were scored to give an overall quality score (OQS, range 0–13), and a combined key methodological index score (MIS) (range 0–5) of five key methodological items was measured. The OQS of 17 items from the STRICTA guideline (range 0–17) was also measured.

**Results:**

In total, 26 reports were evaluated. The median OQS based on the CONSORT guideline was 8 (minimum 5, maximum 11), and “trial design,” “sample size,” “ancillary analyses,” and “harms” had a positive rate of less than 10%. The median MIS was 2 (minimum 0, maximum 5), with “allocation concealment and implementation,” “blinding,” and “intent-to-treat analysis” having a positive rate of less than 15%. The median OQS based on the STRICTA guideline was 12 (minimum 8, maximum 14), with “extent to which treatment was varied (1c),” “number of needle insertions per subject per session (2a),” and “setting and context of treatment (4b)” having a positive rate of less than 10%.

**Conclusions:**

The overall quality of reports on RCTs of SA treatment for VD was moderate to low. The quality of methodological items was markedly lower than that of other items. The CONSORT and STRICTA guidelines should be used more frequently to standardize the quality of RCT reports of SA treatment for VD.

## Background

Vascular dementia is the second most common cause of dementia after that caused by Alzheimer’s disease [1–3]. It is thought to result from cognitive impairment caused by changes resulting from cerebrovascular disorders [4] and from various outcomes of ischemic and hemorrhagic encephalopathy that induce mental and physical disabilities [5].

According to a report from the World Health Organization (WHO), there were currently estimated to be 35.6 million people with dementia worldwide in 2012, which will double by 2030 and more than triple by 2050 [6]. Dementia is a challenge to patients, caregivers, and healthcare providers [7] and carries a heavy financial burden, with the annual cost of care per patient ranging from 17,000 to 55,200 US$ for serve dementia [8].

Scalp acupuncture therapy stimulates the lesion area on the scalp to produce therapeutic effects. Although this treatment modality has been performed for more than 1000 years, the treatment has advanced in recent years [9], and the WHO published the Standard International Acupuncture Nomenclature in 1991 [10]. The efficacy of scalp acupuncture for vascular dementia has been confirmed by numerous empirical clinical studies [11–13]. However, a more reliable scientific method should be developed to validate the effectiveness of scalp acupuncture for vascular dementia.

Some researchers have implemented randomized controlled trials (RCTs) as a means to validate the therapeutic effects and efficacy of acupuncture, and this study design is considered optimal to assess the effectiveness of an intervention [14]. However, despite the effective design of an RCT, inappropriate study methods can affect the reliability and validity of outcomes [15]. Thus, research methodology is a critical factor that determines overall study quality [16]. Hence, there is an urgent need to assess the quality of RCTs based on systematic quality control and assessment for all stages of clinical trials, from planning and execution to analysis [17].

The Consolidated Standards of Reporting Trials (CONSORT) guideline, which was developed in 2001 and updated in 2010, was intended to assist in the assessment and interpretation of parallel-group RCTs so that users could identify biased outcomes and ultimately improve RCT reporting [18]. The Standards for Reporting Interventions in Controlled Trials of Acupuncture (STRICTA) guideline was designed in 2001 and revised in 2010 to assist in the reporting of clinical studies on acupuncture [19]. The combination of these guidelines helps researchers to assess the completeness and transparency of RCTs [20].

The aim of this study was to evaluate the quality of reports on domestic and foreign RCTs in which scalp acupuncture was used for the treatment of vascular dementia, based on the CONSORT and STRICTA guidelines, in order to provide preliminary data from clinical trials on scalp acupuncture for vascular dementia.

## Methods

### Collection of the RCTs

We searched for all reports about RCTs in which scalp acupuncture was used for the treatment of vascular dementia that were published before the end of December 2015 in eight databases (PubMed, EMbase, Cochrane Library, China National Knowledge Infrastructure, National Institute of Informatics Scholarly and Academic Information Navigator, National Digital Science Library, Korean Traditional Knowledge Portal, and Korean Studies Information Service System). The search terms were “vascular dementia, dementia,” and “scalp acupuncture.”

### Literature selection/exclusion criteria

#### Types of studies

RCTs in which a control group of patients with vascular dementia who received a placebo, sham treatment, or conventional treatment was compared with an experimental group treated with scalp acupuncture were included. Non-randomized, cross-over RCTs, case reports, and case-control studies were excluded.

#### Types of participants

The subjects of RCTs were patients who were diagnosed with vascular dementia based on the *Diagnostic and Statistical Manual of Mental Disorders*, *Fourth Edition*, the U.S. National Institute of Neurological Disease and Stroke and the Association Internationale pour La Recherche et l'Enseignement en Neurosciences, or definitively via computed tomography or magnetic resonance imaging, regardless of age, sex, and other demographic factors.

#### Types of intervention groups

The types of scalp acupuncture interventions included were body electro-acupuncture and body acupuncture, accompanied by drug therapy using Western or Chinese medicine or physical therapy such as exercise and rehabilitation.

### Assessment of the quality of the reports

This study assessed the quality of the reports based on the 25-item CONSORT 2010 guideline and the 6-item STRICTA 2010 guideline. Each item on the checklists detailed in these reports was answered with a “yes” or “no.” Two raters independently performed the assessments with reference to the explanation provided by the CONSORT guideline and STRICTA guideline. Their assessments were compared, and any discrepancies were determined by consensus with the third author [21, 22].

#### Rating of overall reporting quality

Each of 13 items included in the CONSORT guideline was scored to compute an overall quality score (OQS) (range 0–13) (Table 1) [23–25]. The discussion section items of the CONSORT guideline were excluded, due to the difficulty of objectively evaluating them [26–28]. Each of 17 items in the STRICTA guideline was also scored (range 0–17) (Table 2). For scoring of the quality of items, 1 point was given if the information for each item was stated in the study, and 0 points if the information was not stated or was unclear [19, 29].

**Table 1:** Rating of overall quality using items from the CONSORT guideline (n = 26)

Item	Criteria	Description	Number of positive trials^a^	Percentage	Cohen’s *к* coefficient	95% CI
1	“Randomized” in the title or abstract	Study identified as a randomized controlled in the title or abstract	25	96	1.00	1.00
2	Background	Adequate description of the scientific background and explanation of rationale	14	54	0.68	0.39 to 0.97
3	Trial design	Description of trial design (such as parallel, factorial) including allocation ratio	2	8	1.00	1.00
4	Participants	Description of the eligibility criteria for participants	26	100	1.00	1.00
5	Interventions	Details of the interventions intended for each group	26	100	1.00	1.00
6	Outcomes	Definition of primary (and secondary when appropriate) outcome measures	19	73	0.78	0.49 to 1.07
7	Sample size	Description of sample size calculation	1	4	0.65	-0.03 to 1.32
12	Statistical methods	Description of the statistical methods used to compare groups for primary outcomes, subgroup analyses, or adjusted analyses	23	88	0.78	0.36 to 1.20
13	Flow chart	Details on the flow of participants through each stage of the trials (number of patients randomly assigned, receiving intended treatment, completing the protocol and analyzed)	22	85	0.62	0.12 to 1.12
14	Recruitment	Dates defining the periods of recruitment and follow up	21	81	0.71	0.32 to 1.10
17	Outcomes and estimation	For each primary and secondary outcome, a summary of results for each group is given, and the estimated effect size and its precision (for example, 95% CI)	20	77	0.75	0.42 to 1.08
18	Ancillary analyses	Clear statement of whether subgroup/adjusted analyses were prespecified or exploratory	0	0	1.00	1.00
19	Harms	Description of all important adverse events in each group	2	8	0.63	0.13 to 1.12

*CONSORT* Consolidated Standards of Reporting Trials. ^a^Positive trials, the information for each item was stated so 1 point was given

**Table 2:** Rating of overall quality using items from the STRICTA guideline (n = 26)

Item	Criteria	Description	Number of positive trials^a^	Percentage	Cohen’s *к* coefficient	95% CI
1	Acupuncture rationale	(1a) Style of acupuncture (e.g., traditional Chinese medicine, Japanese, Korean, Western medical, five element, ear acupuncture, etc.)	26	100	1.00	1.00
		(1b) Reasoning for treatment provided, based on historical context, literature sources and/or consensus methods, with references where appropriate	22	85	0.84	0.52 to 1.15
		(1c) Extent to which treatment was varied	1	4	1.00	1.00
2	Details of needling	(2a) Number of needle insertions per subject per session (mean and range where relevant)	2	8	1.00	1.00
		(2b) Names (or location if no standard name) of points used (uni-/bilateral)	26	100	1.00	1.00
		(2c) Depth of insertion, based on a specified unit of measurement or on a particular tissue level	17	65	0.72	0.43 to 1.02
		(2d) Responses sought (e.g., *de qi* or muscle twitch response)	20	77	0.88	0.66 to 1.10
		(2e) Needle stimulation (e.g., manual or electrical)	13	50	0.52	0.19 to 0.86
		(2f) Needle retention time	21	81	0.87	0.61 to 1.12
		(2 g) Needle type (diameter, length and manufacturer or material)	22	85	0.84	0.52 to 1.15
3	Treatment regimen	(3a) Number of treatment sessions	23	88	0.78	0.36 to 1.20
		(3b) Frequency and duration of treatment sessions	25	96	1.00	1.00
	Other components of treatment	(4a) Details of other interventions administered to the acupuncture group (e.g., moxibustion, cupping, herbs, exercises, lifestyle advice)	15	58	0.67	0.37 to 0.97
		(4b) Setting and context of treatment, including instructions to practitioners, and information and explanations to patients	1	4	0.65	-0.03 to 1.32
5	Practitioner background	(5) Description of participating acupuncturists (qualification or professional affiliation, years in acupuncture practice, other relevant experience)	22	77	0.74	0.43 to 1.08
6	Control or comparator interventions	(6a) Rationale for the control or comparator in the context of the research question, with sources that justify the choice(s)	9	35	0.76	0.51 to 1.02
		(6b) Precise description of the control or comparator. If sham acupuncture or any other type of acupuncture-like control is used, provide details as for items 1–3 above	26	100	1.00	1.00

*STRICTA* Standards for Reporting Interventions in Controlled Trials of Acupuncture. ^a^Positive trials, the information for each item was stated so 1 point was given

### Rating of key methodological items

In the CONSORT guideline, the key items pertaining to methodology (i.e., “randomization,” “allocation concealment,” “blinding,” “baseline characteristics,” and “intention-to-treat (ITT) analysis”) were assessed separately as they were related to potential factors of bias [30–32]. A combined key methodological index score (MIS) (range 0–5) of these five key methodological items was measured (Table 3). For each of the studies, 1 point was given when the reports had information on each item, and 0 points were given if the information was not stated or was unclear.

**Table 3:** Quality of key methodological items (n = 26)

Item	Criteria	Description	Number of positive trials^a^	%	Cohen’s *к* coefficient	95% CI
8	Randomization	Description of the method used to generate the random sequence	14	54	0.68	0.39 to 0.97
9 and 10	Allocation concealment and implementation	Description of the method used to implement the random allocation sequence assuring the concealment until interventions are assigned	3	12	0.84	0.52 to 1.15
11	Blinding	Whether or not participants, those administering the interventions, or those assessing the outcomes were blinded to group assignment	3	12	1.00	1.00
15	Baseline data	An outline of baseline demographic and clinical characteristics of each group	11	42	0.92	0.77 to 1.07
16	Intent-to-treat analysis	No. of participants in each group included in each analysis and whether it was done by “intention to treat”	3	12	0.71	0.32 to 1.10

^a^Positive trials, the information for each item was stated so 1 point was given

### Data extraction and analysis

Two raters independently performed the assessments with reference to a discussion about the definitions of each item and the instructions provided in the CONSORT and STRICTA guidelines. Their assessments of the reports were compared, and any discrepancies were resolved by consensus with the third author.

Cohen’s *к*-statistic was quantified to assess the inter-rater agreement. We defined agreement of 0.20 as “poor,” that greater than 0.20 but less than 0.40 as “low,” that greater than 0.40 but less than 0.60 as “moderate,” that greater than 0.60 but less than 0.80 as “substantial,” that greater than 0.80 as “good,” and agreement of 1 as “perfect” [23]. Items upon which there was any disagreement were specifically examined to reach an agreement. Cohen’s *к*-statistic analysis was performed using SAS software, version 9.3 (SAS Institute, Inc., Cary, NC, USA).

In order to evaluate the overall quality of reported RCTs and relevant factors, OQS was used as a dependent variable modeled using linear regression. Only variables with *p* ≤ 0.10 on univariate analysis were included in the multivariate regression model to identify significant variables (*p* ≤ 0.05). To analyze the factors related to methodological quality, the MIS was used as an outcome variable in regression analysis. Linear and ordinal regression analysis was performed using SPSS software version 20.0 (SPSS, Chicago, IL, USA).

## Results

**Fig. 1:**
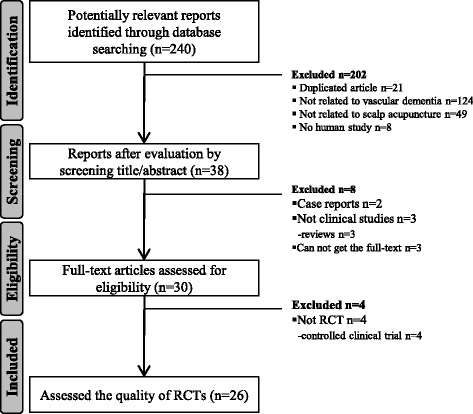
Flowchart of the article selection process. *RCT* randomized controlled trial

The RCT selection process for this study is depicted in Fig. 1. After identification, screening, and determining the eligibility of the reports, a total of 26 relevant RCTs were included in the final analysis.

### Features of the included RCTs

**Fig. 2:**
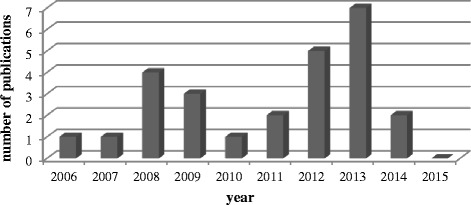
Number of publications

A number of RCTs on scalp acupuncture for vascular dementia were reported in the last 10 years. Most of these studies were reported in the last 8 years, with one in 2006 (3.8%), one in 2007 (3.8%), four in 2008 (15.4%), three in 2009 (11.5%), one in 2010 (3.8%), two in 2011 (7.7%), five in 2012 (19.2%), seven in 2013 (26.9%), two in 2014 (7.7%), and none in 2015 (0%) (Fig. 2).

Twenty-two reports were written in Chinese (84.6%) and four reports were in English (15.4%), but all of the 26 reports were published in China by Chinese researchers. With regard to the types of interventions in the control group, among the 26 RCTs evaluated, Western medicine was used in 20 studies (76.9%), body electro-acupuncture was used in three studies (11.5%), and body acupuncture was used in one study (3.8%), with a sample size ranging from 40 to 241 subjects. Twelve studies were conducted with external funding (46.2%).

### Quality of the reports

#### Rating of overall quality

Table 1 shows the ratings of overall quality based on the CONSORT guideline. The median OQS of the 26 RCTs was 8 (minimum 5, maximum 11). The items that were relatively more neglected in the reports were “trial design,” “sample size,” “ancillary analyses,” and “harms,” showing less than 10% of a positive rate. The two raters reached a substantial (items 2, 6, 7, 12, 13, 14, 17, and 19), perfect agreement (items 1, 3, 4, 5, and 18) for all items with the exception of “outcomes and estimation” (Table 1).

Table 2 shows the ratings of overall quality based on the STRICTA guideline. The median OQS of the 26 RCTs was 12 (minimum 8, maximum 14). Items that were relatively neglected by RCTs were “extent to which treatment was varied (1c),” “number of needle insertions per subject per session (2a),” and “setting and context of treatment (4b),” with less than 10% of a positive rate. The two raters reached substantial (items 2c, 3a, 4a, 4b, 5, and 6a), good, or perfect agreement (items 1a, 1b, 1c, 2a, 2b, 2d, 2f, 2 g, 3b, and 6b) for all items with the exception of “needle stimulation (2e)” (Table 2).

#### Rating of key methodological items

The median MIS value for the five items (items 8, 9, 10, 11, 15, and 16) was 2 (minimum 0, maximum 5). Particularly, “allocation concealment and implementation,” “blinding,” and “intent-to-treat analysis” had less than 15% of a positive rate (Table 3). The two raters reached substantial (items 8 and 16), good (items 9, 10, and 15), or perfect agreement (item 11) for all items (Table 3).

#### Exploratory analysis: factors associated with better quality of the reports

In a single-factor analysis of variance, all the factors related to the CONSORT and STRICTA guidelines were found to have no mutual association. Ordinal regression analysis showed that the variables to enhance the quality of the methodology were not associated with the MIS (*p* > 0.05).

## Discussion

In this study, a comprehensive and systematic search of domestic and foreign RCTs on scalp acupuncture for the treatment of vascular dementia was performed, and the quality of these reports was assessed based on the CONSORT and STRICTA guidelines. These two guidelines are useful tools that were developed to identify gaps in the reporting of RCTs and have been widely used to assess the quality of reports on acupuncture studies [20].

Based on the CONSORT guideline, the median OQS of the 26 RCTs was 8, which was greater than 50% of the total score, and that of the STRICTA items was 12, which was greater than 70% of the total score. However, the “trial design,” “sample size,” “ancillary analyses,” and “harms” items of the CONSORT guideline and the “extent to which treatment was varied (1c),” “number of needle insertions per subject per session (2a),” and “setting and context of treatment (4b)” items of the STRICTA guideline were inappropriately addressed or neglected in most of the reports. This finding is in line with the results of other quality assessment studies [20, 25, 29, 30].

It is important for researchers to clearly state the design of clinical trial studies (randomized, parallel, double-blind, placebo-controlled, sham-controlled, etc.), including the details of the randomization, as indicated in the “trial designs” item of the CONSORT guideline. Even in cases of a parallel randomization ratio such as 8:8 for two groups, it is important to clearly provide the allocation ratio to improve the quality of reporting. The importance of trial design is especially highlighted among non-universal clinical trials with large sample sizes or complex analyses [31].

Sample size calculation is required to maintain a balance between the statistical considerations and the differences in therapeutic effects for intervention groups. A clinically significant difference between the intervention group and the control group generates reliable outcomes if the sample size is sufficiently large [20]. However, it may be difficult to achieve the planned sample size because recruiting qualified subjects within a limited time period is challenging [33, 34]. RCTs with a small sample size are often prone to biases or are insufficient in measuring the therapeutic advantages [26]. Therefore, researchers should pay more attention to sample size measurements by consulting with professional clinical statisticians in order to identify highly reliable and significant differences between control and intervention groups.

With regard to the “ancillary analyses” item, performing multiple analyses of the same data may introduce biases that lead to exaggeration in the interpretation of the study results [35], and reporting of analytical outcomes that were not prespecified in the research protocol leads to selective reporting bias for subgroup analyses [36]. Therefore, authors should report the results of prespecified analyses for high reliability, and clearly state the reason for and purpose of conducting subgroup analyses.

A randomized trial is the optimal design to generate efficacy and safety data, but it is difficult to detect rarely occurring “harms” through such a design. Many RCTs have provided inappropriate reports of abnormal responses [37] or low-quality reports of abnormal responses [38]. Furthermore, very few studies mention severe abnormal responses or subjects who dropped out because of abnormal responses [39]. Information about the risk and the benefits of an intervention is required to help the participants of clinical trials make reasonable and balanced decisions. The occurrence and characteristics of abnormal responses affect the acceptance and usefulness of a particular intervention [20].

The “extent to which treatment was varied (1c)” item had a 3% of a positive rate in this study, which is similar to the results of a previous study [22]. Variations between treatments in each clinical trial should be minimized by using a standardized protocol, and researchers should describe the degree of personalized treatment to both the patient and practitioner [40]. The “number of needle insertions per subject per session (2a)” item had an 8% positive rate in this study. Researchers should strictly report the total number of needles used and as many other related details as possible [40]. The “setting and context of treatment (4b)” item is an additional important component to report [41]. As changes in medical practice or context of treatment of the patients affect the outcome of experiments [29], researchers must report information that is provided to the patients about the interventions for treatment and control groups [20].

The median MIS, which represents the quality of methodological reports of the CONSORT guideline, was markedly low at 2, and the majority of studies had insufficient or missing information on “allocation concealment and implementation,” “blinding,” and “intent-to-treat analysis.” These key methodological items play an important role in preventing selection bias, performance/detection bias, and attribution bias [42]. An inappropriate study design could lead to exaggeration of the clinical outcome [23, 24, 43, 44], which has been reported in a few other quality assessment studies [45, 46]. In reports of RCTs of Chinese medicine, the quality of the reporting of the methodology is poor, warranting the use of CONSORT guidelines. We recommend that clinical researchers should be professionally trained with regard to the design and reporting of RCTs, and that the studies should be stringently peer-reviewed to improve the overall quality of research articles for their submission and publication in international journals.

Although we have comprehensively and systematically assessed 26 RCTs, there are a few limitations in our study. First, it was difficult to search for studies published in languages other than English and Chinese, such as those published in Japanese or other Asian languages. Nevertheless, most of the studies on scalp acupuncture treatment are published in Chinese or English. Second, only articles published between 2006 and 2015 were included in this study, which may be associated with the increased incidence of geriatric diseases caused by an upsurge of the elderly population in the last 10 years.

## Conclusions

This study confirmed the quality of reporting of RCTs on scalp acupuncture for the treatment of vascular dementia. The CONSORT and STRICTA guidelines should be more widely used to enhance the quality of the reporting of RCTs in the future. The present study findings suggest that more reliable research on scalp acupuncture for the treatment of vascular dementia, including larger sample sizes, is warranted in the future.

## Abbreviations

CONSORT: Consolidated Standards of Reporting Trials
ITT analysis: Intention-to-treat analysis
MIS: Methodological index score
OQS: Overall quality score
RCT: Randomized controlled trial
STRICTA: Standards for Reporting Interventions in Controlled Trials of Acupuncture
WHO: World Health Organization
